# Predictive values of lung cancer alarm symptoms in the general population: a nationwide cohort study

**DOI:** 10.1038/s41533-020-0173-3

**Published:** 2020-04-07

**Authors:** Peter F. Haastrup, Dorte E. Jarbøl, Kirubakaran Balasubramaniam, Lisa M. S. Sætre, Jens Søndergaard, Sanne Rasmussen

**Affiliations:** Research Unit of General Practice, Department of Public Health, University of Southern Denmark J.B., Winsløws Vej 9a, DK-5000 Odense C, Denmark

**Keywords:** Diagnosis, Respiratory signs and symptoms, Respiratory signs and symptoms, Diagnosis, Respiratory signs and symptoms

## Abstract

We aimed to firstly determine the 1-year predictive values of lung cancer alarm symptoms in the general population and to analyse the proportion of alarm symptoms reported prior to diagnosis, and secondly analyse how smoking status and reported contact with general practitioners (GPs) regarding lung cancer alarm symptoms influence the predictive values. The study was a nationwide prospective cohort study of 69,060 individuals aged ≥40 years, randomly selected from the Danish population. Using information gathered in a survey regarding symptoms, lifestyle and healthcare-seeking together with registry information on lung cancer diagnoses in the subsequent year, we calculated the predictive values and likelihood ratios of symptoms that might be indicative of lung cancer. Furthermore, we analysed how smoking status and reported contact with GPs regarding the alarm symptoms affected the predictive values. We found that less than half of the patients had reported an alarm symptom six months prior to lung cancer diagnosis. The positive predictive values of the symptoms were generally very low, even for patients reporting GP contact regarding an alarm symptom. The highest predictive values were found for dyspnoea, hoarseness, loss of appetite and for current heavy smokers. The negative predictive values were high, all close to 100%. Given the low positive predictive values, our findings emphasise that diagnostic strategies should not focus on single, specific alarm symptoms, but should perhaps focus on different clusters of symptoms. For patients not experiencing alarm symptoms, the risk of overlooking lung cancer is very low.

## Introduction

Many countries have implemented referral guidelines for patients with respiratory alarm symptoms in order to expedite diagnosis of lung cancer and reduce the diagnostic interval, thus increasing survival rates^1^. Presentation of alarm symptoms in primary care settings is often preceded by patients’ recognition of potentially serious symptoms. Qualitative studies have demonstrated that patients with lung cancer often experience symptoms months before diagnosis, but do not interpret such symptoms as serious enough to warrant seeking health care^2^. Furthermore, public awareness of lung cancer symptoms is low, and public knowledge about risk factors other than smoking is sparse^3^. Therefore, many health campaigns have been conducted with the aim of increasing public awareness of these alarm symptoms as indicative of cancer in hopes of reducing the interval before lung cancer diagnosis^4,5^. Despite awareness that smoking is an important risk factor for lung cancer, smokers are less likely to seek medical attention when experiencing respiratory alarm symptoms^6^.

In order to identify early signs of lung cancer, retrospective surveys of symptom experiences prior to diagnosis have revealed prolonged cough, dyspnoea and hemoptysis as key respiratory alarm symptoms^7^. However, such symptoms also often precede common, more benign conditions; on the other hand, many lung cancer patients experience both tumour-specific symptoms such as coughing and dyspnoea as well as systemic, nonspecific symptoms of malignancy such as weight loss and loss of appetite^8,9^. Based on the data from a nationwide survey of symptom experiences in the general population, we have analysed the prevalence of several symptoms^10–13^ as well as the predictive value of gastrointestinal alarm symptoms^14,15^. Lung cancer is one of the most common worldwide, causing high morbidity and mortality. The prognosis of lung cancer is highly dependent on stage of disease at the time of diagnosis, and since many patients are diagnosed at advanced stages, the survival rates of lung cancer are poor^16^. The literature examining predictive values of specific and nonspecific symptoms of lung cancer is, however, sparse, and prospective studies that systematically record such symptoms and explore their predictive values for lung cancer diagnosis are needed^2,17^. We therefore conducted this study, aiming to (1) assess lung cancer alarm symptoms reported by individuals prior to lung cancer diagnosis, and (2) analyse the predictive values of lung cancer alarm symptoms experienced in the general population. Furthermore, our aim was to analyse the association between experiencing single or multiple respiratory alarm symptoms and receiving a lung cancer diagnosis within 6 months or 12 months, and also to analyse how smoking status and reported contact with general practitioners (GPs) regarding lung cancer alarm symptoms influence predictive values of the symptoms.

## Results

**Fig. 1:**
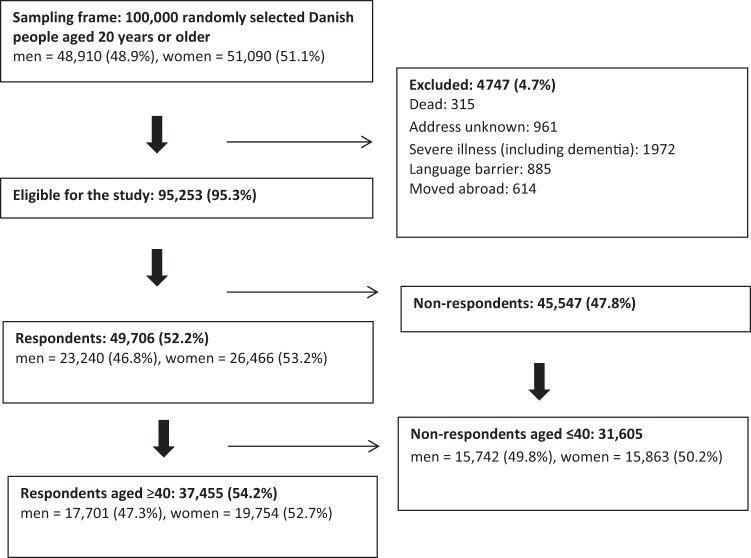
Study cohort. Flow chart of the study cohort selection process.

A total of 4747 (4.7%) of the 100,000 individuals invited to answer the questionnaire were not eligible because they were deceased, unknown address, severe illness, language barrier or emigration. A total of 95,253 subjects were eligible for the study, of whom 69,060 were ≥40 years. Among the eligible individuals ≥40 years, 37,455 (54.2%) completed the questionnaire. A total of 47.3% (17,701) of respondents age 40 years or older were male (Fig. 1).

We found that 6,261 (16.7%) of respondents ≥40 years reported at least one specific alarm symptom. The most frequently reported symptom was cough lasting more than 4 weeks, which was reported by 3,400 (9.1%) of the respondents (Table 1).

**Table 1:** Prevalence of symptoms, smoking status and GP contact among respondents 40 years and older.

	*N* (%)
Total	37,455 (100.0%)
Age	
40–59 years	20,305 (54.2%)
60–74 years	14,018 (37.4%)
75 years	3132 (8.4%)
Sex	
Female	19,754 (52.7%)
Male	17,701 (47.3%)
Specific respiratory alarm symptoms	
Cough (>4 weeks)	3400 (9.1%)
Dyspnoea	3080 (8.2%)
Hoarseness (>4 weeks)	1404 (3.7%)
Hemoptysis	44 (0.1%)
At least one specific alarm symptom	6261 (16.7%)
Number of respiratory alarm symptoms	
0	31,194 (83.3%)
1	4826 (12.9%)
≥2	1435 (3.8%)
Nonspecific alarm symptoms	
Weight loss	973 (2.6%)
Feeling unwell	4321 (11.5%)
Tiredness	16,282 (43.5%)
Loss of appetite	1796 (4.8%)
Lack of energy	12,618 (33.7%)
Smoking status	
Smoker, current	7506 (20.0%)
Smoker, current, heavy smoker	2917 (7.8%)
Smoker, current, light smoker	4322 (11.5%)
Not currently smoking or unknown	29,949 (80.0%)
At least one specific symptom and smoking	1917 (5.1%)
GP contact regarding at least one specific symptom	2764 (7.4%)
GP contact regarding at least one specific symptom and current smoker	667 (1.8%)

Our analyses of the socioeconomic characteristics of eligible individuals aged ≥40 demonstrate that respondents were younger and with higher rates of cohabitation and labour market affiliation, higher educational and income levels, and were more often of Danish ethnicity when compared with non-respondents. The details of the analyses are reported elsewhere^15^. A total of 20% of the respondents stated that they were daily smokers. In a national health survey from 2013^18^, 17% of the population were daily smokers, indicating that the smoking behaviour of our respondents was comparable with the general population.

The number of incident lung cancer cases among the respondents ≥40 years was 22 after 6 months (0.58‰) and 41 after 12 months (1.1‰). Among non-respondents, a total of 27 new cases of lung cancer were registered after 6 months (0.85‰) and 58 new cases after 12 months (1.83‰).

The PPV for being diagnosed with lung cancer in the 6-month follow-up period was 0.2 (95% CI: 0.1–0.3) for individuals reporting at least one of the specific alarm symptoms. The PPV was similar in the 12-month follow-up period. Among the specific alarm symptoms, the highest PPVs were found for dyspnoea, with a PPV of 0.2 (95% CI: 0.1–0.4) and LR+ of 2.8 (95% CI: 1.3–6.0) in the 6-month follow-up (Table 2), and hoarseness, with a PPV of 0.3 (95% CI: 0.1–0.7) and LR+ of 2.6 (95% CI: 1.0–6.6) in the 12-month follow-up (Table 3). Among the nonspecific symptoms, the highest PPVs were found for loss of appetite, with a PPV of 0.3 (95% CI: 0.1–0.6) and LR+ of 4.8 (95% CI: 2.2–10.3) in the 6-month follow-up (Table 2), and a PPV of 0.3 (95% CI: 0.1–0.6) and LR+ of 2.5 (95% CI: 1.1–5.8) in the 12-month follow-up (Table 3).

**Table 2:** Positive and negative predictive values (PPV and NPV) and positive and negative likelihood ratios (LR+ and LR−) for lung cancer diagnosis in six-month follow-up period after study questionnaire.

	*N* (%)	PPV	(95% CI)	LR+	(95% CI)	NPV	(95% CI)	LR−	(95% CI)
Age									
40–59 years	7 (31.8%)	0.0	(0.0; 0.1)	0.6	(0.3; 1.1)	99.9	(99.9; 100.0)	1.5	(1.1; 2.0)
60–74 years	10 (45.5%)	0.1	(0.0; 0.1)	1.2	(0.8; 1.9)	99.9	(99.9; 100.0)	0.9	(0.6; 1.3)
75 years	5 (22.7%)	0.2	(0.1; 0.4)	2.7	(1.3; 5.9)	100.0	(99.9; 100.0)	0.8	(0.7; 1.1)
Sex									
Female	11 (50.0%)	0.1	(0.0; 0.1)	0.9	(0.6; 1.4)	99.9	(99.9; 100.0)	1.1	(0.7; 1.6)
Male	11 (50.0%)	0.1	(0.0; 0.1)	1.1	(0.7; 1.6)	99.9	(99.9; 100.0)	0.9	(0.6; 1.4)
Specific respiratory alarm symptoms									
Cough (>4 weeks)	5 (22.7%)	0.1	(0.0; 0.3)	2.5	(1.2; 5.4)	100.0	(99.9; 100.0)	0.8	(0.7; 1.1)
Dyspnoea	5 (22.7%)	0.2	(0.1; 0.4)	2.8	(1.3; 6.0)	100.0	(99.9; 100.0)	0.8	(0.7; 1.1)
Hoarseness (>4 weeks)	≤3^a^								
Hemoptysis	≤3^a^								
At least one specific alarm symptom	10 (45.5%)	0.2	(0.1; 0.3)	2.7	(1.7; 4.3)	100.0	(99.9; 100.0)	0.7	(0.4; 1.0)
Number of respiratory alarm symptoms									
0	12 (54.5%)	0.0	(0.0; 0.1)	0.7	(0.4; 1.0)	99.8	(99.7; 99.9)	2.7	(1.7; 4.3)
1	6 (27.3%)	0.1	(0.0; 0.3)	2.1	(1.1; 4.2)	100.0	(99.9; 100.0)	0.8	(0.6; 1.1)
≥2	4 (18.2%)	0.3	(0.1; 0.7)	4.8	(2.0; 11.6)	100.0	(99.9; 100.0)	0.9	(0.7; 1.0)
Nonspecific alarm symptoms									
Weight loss	≤3^a^								
Feeling unwell	6 (27.3%)	0.1	(0.1; 0.3)	2.4	(1.2; 4.7)	100.0	(99.9; 100.0)	0.8	(0.6; 1.1)
Tiredness	13 (59.1%)	0.1	(0.0; 0.1)	1.4	(1.0; 1.9)	100.0	(99.9; 100.0)	0.7	(0.4; 1.2)
Loss of appetite	5 (22.7%)	0.3	(0.1; 0.6)	4.8	(2.2; 10.3)	100.0	(99.9; 100.0)	0.8	(0.6; 1.0)
Lack of energy	9 (40.9%)	0.1	(0.0; 0.1)	1.2	(0.7; 2.0)	99.9	(99.9; 100.0)	0.9	(0.6; 1.3)
Smoking status									
Smoker, current	10 (45.5%)	0.1	(0.1; 0.2)	2.3	(1.4; 3.6)	100.0	(99.9; 100.0)	0.7	(0.5; 1.0)
Smoker, current, heavy smoker	4 (18.2%)	0.1	(0.0; 0.4)	2.3	(1.0; 5.7)	99.9	(99.9; 100.0)	0.9	(0.7; 1.1)
Smoker, current, light smoker	6 (27.3%)	0.1	(0.1; 0.3)	2.4	(1.2; 4.7)	100.0	(99.9; 100.0)	0.8	(0.6; 1.1)
Not currently smoking or unknown	12 (54.5%)	0.0	(0.0; 0.1)	0.7	(0.5; 1.0)	99.9	(99.8; 99.9)	2.3	(1.4; 3.6)
At least one specific symptom and smoking	≤3^a^								
GP contact regarding at least one specific symptom	4 (18.2%)	0.1	(0.0; 0.4)	2.5	(1.0; 6.0)	99.9	(99.9; 100.0)	0.9	(0.7; 1.1)
GP contact regarding at least one specific symptom and current smoker	≤3^a^								

^a^Due to Danish legislation, reporting of the data for a number of patients ≤3 is not permitted.

**Table 3:** Positive and negative predictive values (PPV and NPV) and positive and negative likelihood ratios (LR+ and LR−) for lung cancer diagnosis in 12-month follow-up period after study questionnaire.

	*N*(%)	PPV	(95% CI)	LR+	(95% CI)	NPV	(95% CI)	LR−	(95% CI)
Age									
40–59 years	9 (22.0%)	0.0	(0.0; 0.1)	0.4	(0.2; 0.7)	99.8	(99.7; 99.9)	1.7	(1.4; 2.0)
60–74 years	21 (51.2%)	0.1	(0.1; 0.2)	1.4	(1.0; 1.8)	99.9	(99.9; 99.9)	0.8	(0.6; 1.1)
75 years	11 (26.8%)	0.4	(0.2; 0.6)	3.2	(1.9; 5.3)	99.9	(99.9; 99.9)	0.8	(0.7; 1.0)
Sex									
Female	20 (48.8%)	0.1	(0.1; 0.2)	0.9	(0.7;1.3)	99.9	(99.8; 99.9)	1.1	(0.8; 1.5)
Male	21 (51.2%)	0.1	(0.1; 0.2)	1.1	(0.8;1.5)	99.9	(99.8; 99.9)	0.9	(0.7; 1.3)
Specific respiratory alarm symptoms									
Cough (>4 weeks)	6 (14.6%)	0.2	(0.1; 0.4)	1.6	(0.8; 3.4)	99.9	(99.9; 99.9)	0.9	(0.8; 1.1)
Dyspnoea	7 (17.1%)	0.2	(0.1; 0.5)	2.1	(1.1; 4.1)	99.9	(99.9; 99.9)	0.9	(0.8; 1.0)
Hoarseness (>4 weeks)	4 (9.8%)	0.3	(0.1; 0.7)	2.6	(1.0; 6.6)	99.9	(99.9; 99.9)	0.9	(0.8; 1.0)
Hemoptysis	≤3^a^								
At least one specific alarm symptom	13 (31.7%)	0.2	(0.1; 0.4)	1.9	(1.2;3.0)	99.9	(99.9; 99.9)	0.8	(0.7; 1.0)
Number of respiratory alarm symptoms									
0	28 (68.3%)	0.1	(0.1; 0.1)	0.8	(0.7; 1.0)	99.8	(99.6; 99.9)	1.9	(1.2; 3.0)
1	8 (19.5%)	0.2	(0.1; 0.3)	1.5	(0.8; 2.8)	99.9	(99.9; 99.9)	0.9	(0.8; 1.1)
≥2	5 (12.2%)	0.3	(0.1; 0.8)	3.2	(1.4; 7.3)	99.9	(99.9; 99.9)	0.9	(0.8; 1.0)
Nonspecific alarm symptoms									
Weight loss	≤3^a^								
Feeling unwell	6 (14.6%)	0.1	(0.1; 0.3)	1.3	(0.6; 2.7)	99.9	(99.9; 99.9)	1.0	(0.9; 1.1)
Tiredness	21 (51.2%)	0.1	(0.1; 0.2)	1.2	(0.9; 1.6)	99.9	(99.9; 99.9)	0.9	(0.6; 1.2)
Loss of appetite	5 (12.2%)	0.3	(0.1; 0.6)	2.5	(1.1; 5.8)	99.9	(99.9; 99.9)	0.9	(0.8; 1.0)
Lack of energy	13 (31.7%)	0.1	(0.1; 0.2)	0.9	(0.6; 1.5)	99.9	(99.8; 99.9)	1.0	(0.8; 1.3)
Smoking status									
Smoker, current	15 (36.6%)	0.2	(0.1; 0.3)	1.8	(1.2; 2.7)	99.9	(99.9; 99.9)	0.8	(0.6; 1.0)
Smoker, current, heavy smoker	9 (22.0%)	0.3	(0.1; 0.6)	2.8	(1.6; 5.0)	99.9	(99.9; 99.9)	0.8	(0.7; 1.0)
Smoker, current, light smoker	6 (14.6%)	0.1	(0.1; 0.3)	1.3	(0.6; 2.7)	99.9	(99.9; 99.9)	1.0	(0.9; 1.1)
Not currently smoking or unknown	26 (63.4%)	0.1	(0.1; 0.1)	0.8	(0.6; 1.0)	99.8	(99.7; 99.9)	1.8	(1.2; 2.7)
At least one specific symptom and smoking	≤3^a^								
GP contact regarding at least one specific symptom	4 (9.8%)	0.1	(0.0; 0.4)	1.3	(0.5; 3.4)	99.9	(99.9; 99.9)	1.0	(0.9; 1.1)
GP contact regarding at least one specific symptom and current smoker	≤3^a^								

^a^Due to Danish legislation, reporting of data for a number of patients ≤3 is not permitted.

Contacting a GP regarding at least one of the specific symptoms had a PPV of 0.1 (95% CI: 0.0–0.4). In the 12-month follow-up, being a current heavy smoker carried in itself a PPV of 0.3 (95% CI: 0.1–0.6).

The NPVs were all close to 100, and the LR− values were predominantly close to 1. The highest LR− was 2.7 (95% CI: 1.7–4.3) in the 6-month follow-up for not experiencing an alarm symptom.

## Discussion

In this study, we analysed the predictive values and likelihood ratios of specific and nonspecific alarm symptoms of lung cancer reported by a large sample of the Danish general population ≥40 years. The PPVs were generally very low. Respondents experiencing loss of appetite or dyspnoea or hoarseness for more than 4 weeks had the highest risk of subsequent lung cancer diagnosis. These findings indicate that identifying lung cancer patients based on specific alarm symptoms is challenging, and that nonspecific alarm symptoms could play an important role in identifying individuals at risk.

The NPVs were high, all close to 100, and the highest LR− was for not reporting an alarm symptom. This means that respondents not experiencing any alarm symptoms had a very high chance of not being subsequently diagnosed with lung cancer.

The prospective cohort design is a major strength of this study, as it provides the opportunity to obtain information about pre-diagnostic symptom experiences. The prospective design minimises the risk of recall bias, which is often a substantial challenge in studies of symptoms among cancer patients prior to diagnosis. Identifying lung cancer cases in the Danish Cancer Registry, rather than asking survey respondents, further reduces the risk of recall bias. This registry is based on mandatory data from multiple sources, and is considered a valid source of information on cancer diagnoses^19^.

Another strength is that the study is large-scale and nationwide with a random selection of individuals invited. However, individuals with many symptoms or those who have made contact with their GP multiple times may be more motivated to participate in a survey regarding symptoms and healthcare seeking. This could lead to an overestimation of symptom prevalence. On the other hand, it is plausible that persons experiencing several symptoms and undergoing numerous healthcare visits might not have surplus energy to complete the rather comprehensive questionnaire. Therefore, both over- and underestimation of symptom experiences are possible limitations to our study.

The rather high response rate of 54.2% amongst the eligible individuals ≥40 years is a strength, but it is important to keep in mind that differences between respondents and non-respondents might have affected the results. The number of incident lung cancer cases was higher among non-respondents compared with respondents (1.83‰ vs. 1.1‰). The difference might be explained by socioeconomic disparity between respondents and non-respondents. Low socioeconomic status is a risk factor for lung cancer even when adjusting for smoking status^20,21^: respondents were younger, more often of Danish ethnicity and had higher socioeconomic status than non-respondents. Therefore, the predictive values of lung cancer alarm symptoms reported here might not be generalisable to patients with low socioeconomic status.

We chose to include all incident lung cancer cases in both a 6-month and a 12-month follow-up period after reporting one or more of the respiratory alarm symptoms. We chose so to enhance the likelihood of the symptom being linked to the subsequently diagnosed lung cancer. A longer follow-up period would have increased the number of incident lung cancer cases, but would have also weakened the link between symptom experience and subsequent diagnosis.

A general weakness of surveys is that questionnaires may not measure precisely what they are designed to measure. To ensure that the respondents interpreted the questions and answer categories as intended, we conducted numerous series of validation, pilot testing and field testing prior to survey launch^22^. Based on the results of the pilot testing, it is reasonable to assume that the respondents understood and answered the questions as anticipated. Although the survey comprised questions about symptom experiences within a short time period (the preceding 4 weeks), some memory or recall bias cannot be ruled out.

We chose the lung cancer symptoms based on literature review and symptoms mentioned in lung cancer diagnostic pathways. However, other symptoms and characteristics such as pain or recent pneumonia can be signs of lung cancer as well and could have been included in the analyses. Unfortunately, we did not have access to such information.

Compared with previous studies on symptom prevalence in the general population, we found that the response rate in our study was comparable or even higher^23,24^. Similar to the findings of this study, we found that the PPVs of gastrointestinal alarm symptoms are low in other DaSC studies^14,15^. Compared with a study on respiratory symptoms in a general population in Britain^25^, we found lower rates of cough and dyspnoea. This could probably be explained by the fact that the study by Walabyeki et al. was conducted among individuals aged 50 and older, and had a higher proportion of current smokers (25.8% vs. 20% in our study). Furthermore, their study was carried out between October and March, and our study in June to December^22^. Therefore, a substantial portion of the symptoms recorded in Walabyeki et al. can be assumed to have been caused by seasonal respiratory tract infections^25^.

The most common symptoms of lung cancer are cough, dyspnoea and hemoptysis^26^, but It has been demonstrated that the predictive value of cough, dyspnoea and general symptoms that might be indicative of lung cancer is rather low (0.4–1.1%)^27^. Our study adds to this knowledge, suggesting that similar symptoms in the general population have even lower predictive value. This suggests that individuals deciding to contact a GP regarding a respiratory alarm symptom have a higher risk of underlying lung cancer.

Most lung cancer patients presents with multiple symptoms—both respiratory and constitutional^9^, and in a lung cancer prediction model loss of appetite and having less strength were found the strongest predictors^28^. This is in line with our findings of respondents with nonspecific symptoms, especially loss of appetite, having the highest risk of subsequent lung cancer.

Among the specific alarm symptoms, it has been shown that hemoptysis is the strongest predictor of subsequent lung cancer diagnosis^2,29–31^. However, as observed in our study, hemoptysis is a quite infrequent symptom; due to Danish rules on data protection, we are unable to report data for less than three persons, meaning that we were unable to calculate predictive values for hemoptysis in some categories of analysis.

We found that the PPVs and LR+ for alarm symptoms of lung cancer are quite low in the general population. Further insights could be obtained by focusing in the future on the predictive values of different combinations of specific and nonspecific alarm symptoms and signs. Moreover, combining laboratory results with symptom clusters could provide more information about determinants for cancer. However, this would require inclusion of an even larger population.

Quantification of the diagnostic value of symptoms in lung cancer detection is useful knowledge for clinicians. For the GP reviewing many patient contacts regarding symptoms that might be indicative of lung cancer, it is important knowledge that the risk from respiratory alarm symptoms is low, and that numerous referrals of patients with such symptoms may occur without revealing any cases of lung cancer. It is valuable for the GP to be able to inform patients when referring for lung cancer investigation that even with specific symptoms the risk of lung cancer is very small.

Furthermore, it is noteworthy that nonspecific symptoms such as loss of appetite might be as indicative of lung cancer as specific respiratory symptoms can be. For the clinician, it might be natural to investigate gastrointestinal symptoms in patients reporting loss of appetite, for example; however, our findings suggest that a supplementary investigation of lung cancer may also be worthwhile for such patients. Nonspecific symptoms may trigger the GP’s intuition of serious disease, and our results underline that nonspecific symptoms should be taken seriously. On the other hand, it is also valuable knowledge for GPs that NPVs are high, i.e., there is a very low risk of overlooking lung cancer in the absence of alarm symptoms.

Similarly, our results could be useful for health service planning. As the prognosis for lung cancer depends on stage at diagnosis, a logical intervention to improve survival could be to increase public knowledge of lung cancer symptoms and to encourage visiting a GP. Earlier presentation of symptoms to the GP and earlier referrals for symptom investigation could result in the earlier detection of some cancers^4,5^. However, health service planners need to be aware that as a diagnostic tool, symptoms are not as precise as pathology results due to the subjective and individual nature of symptoms. Given the low predictive value of specific and nonspecific alarm symptoms and the consequences of cancer, both the costs and capacity of the healthcare system should be kept in mind.

Furthermore, the predictive values of nonspecific symptoms demonstrate that referral guidelines and fast-track investigation programs should not be overzealous or based solely on specific respiratory symptoms but possibly also on clusters of symptoms and signs. Referral options should also include patients with nonspecific symptoms according to the GP’s intuition, since this factor is intangible but proven to be a strong predictor of cancer diagnosis^32^.

## Methods

### Study design and population

The study is a part of the Danish Symptom Cohort (DaSC), a nationwide cohort study comprising survey and health register data of a randomly selected cohort of adults in the general population of Denmark^22^. A total of 100,000 adults (≥20 years) randomly selected from the Civil Registration System were invited by mail to participate in a survey about symptom experiences. The letter included a unique log-in for a secure webpage with a comprehensive web-based questionnaire. People with no internet access were offered the survey by telephone interview. The survey was conducted from June to December 2012.

### Questionnaire

The questionnaire comprised items about several symptoms, respondents’ reactions in response to those symptoms, lifestyle factors and general health beliefs and behaviour. This paper comprises the specific and nonspecific lung cancer alarm symptoms (Table 4). The symptoms were selected based on a review of the literature, including national and international guidelines^7,9,17,33^. Other studies derived from DaSC have reported prevalence estimates and predictive values of cancer alarm symptom related to other types of cancer^10,11,13–15^.

**Table 4:** Specific respiratory and nonspecific alarm symptoms.

Specific respiratory alarm symptoms	Nonspecific alarm symptoms
Cough >4 weeks	Weight loss
Dyspnoea	Feeling unwell
Hoarseness >4 weeks	Tiredness
Hemoptysis	Loss of appetite
	Lack of energy

The respondents were asked whether they had experienced one or more of the specific and nonspecific alarm symptoms: “Have you experienced any of the following sensations, symptoms or discomfort within the past 4 weeks?” If respondents confirmed a symptom experience, they were subsequently asked when they had experienced the symptom for the first time. In order to be classified as a respiratory alarm symptom, symptoms of cough and hoarseness had to persist for >4 weeks; other symptoms had to be present within the 4 weeks prior to the survey, but not necessarily for >4 weeks. The respondents were then asked: “Have you contacted your general practitioner concerning the symptom(s) you have experienced within the preceding 4 weeks, by appointment, telephone or e-mail?”

Further information on respondents’ smoking status was obtained through the questionnaire and the respondents were categorised into those who had never smoked, former smokers and current smokers. Current smokers were subdivided into light or heavy smokers: heavy smoking was defined as reported daily tobacco use equivalent to >15 cigarettes. One cheroot was equivalent to three cigarettes, and one cigar or one pipe was equivalent to five cigarettes.

### Register data

Information on sex and age of the invited individuals was obtained through the Civil Registration System. Each individual is assigned a unique identification number at birth or upon obtaining a residency permit in Denmark, thus enabling anonymized, individual-level data to be linked between healthcare registers and socioeconomic registers^34^. This study comprises information from respondents aged 40 or older, due to the extremely low incidence of lung cancer among younger patients^35^.

Diagnoses of lung cancer (ICD-10-CM Code C34 Malignant neoplasm of bronchus and lung) were retrieved from the Danish Cancer Registry^19^, which comprises information about all incident cancer cases in Denmark, including date of diagnosis and ICD-10 codes for malignancy subtype and stage at diagnosis. Only cases of lung cancer diagnosed within a 6-month and 12-month period after questionnaire completion were included in this study. For non-respondents, we also identified the number of lung cancer cases diagnosed within 6- and 12-month periods after invitation to the survey. Both respondent and non-respondent cases were excluded if the individual had been diagnosed with the same cancer (ICD-10 code) within a period of 5 years prior to completion of the questionnaire/invitation.

Data on cohabitation status, educational level, labour market affiliation, income and ethnicity were obtained from nationwide socioeconomic registers^36–38^ to analyse possible socioeconomic disparities between respondents and non-respondents.

### Statistical analysis

The positive predictive value (PPV) for each alarm symptom was calculated by dividing the number of symptomatic respondents subsequently diagnosed with lung cancer by the total number of symptomatic respondents in each category. Negative predictive values (NPVs) were calculated by dividing the number of asymptomatic respondents not subsequently diagnosed with lung cancer by the total number of asymptomatic respondents. The predictive values are presented as percentages. PPVs and NPVs for lung cancer were calculated for each of the symptoms, for at least one of the symptoms, for reported contact with a GP regarding at least one of the symptoms, and reported smoking status. Besides PPVs and NPVs, the positive likelihood ratios (LR+) and negative likelihood ratios (LR−) of the association between symptom experience, GP contact and lung cancer were calculated. These relative ratios were calculated because we expected that the incidence of alarm symptoms would be much higher than the incidence of lung cancer, hence the association between symptom experience and lung cancer would be attenuated by only calculating PPVs and NPVs.

Confidence intervals were calculated using a binomial distribution. All statistical tests used a significance level of *P* < 0.05. Data analysis was conducted using STATA 13.1 statistical software (StataCorp, College Station, TX, USA).

### Ethics approval and consent to participate

The Regional Scientific Ethics Committee for Southern Denmark evaluated the project and concluded that it could be implemented without permission from the Regional Scientific Ethical Committee for Southern Denmark, according to Danish law. Informed consent was obtained from the respondents, and answering the questionnaire was completely voluntary and unpaid. The participants in the study were clearly informed that there would be no clinical follow-up and that they should contact their own GP in case of concern or worry. The project was also approved by the Danish Data Protection Agency (journal number 2011-41-6651).

### Reporting summary

Further information on research design is available in the Nature Research Reporting Summary linked to this article.

## Supplementary information

Reporting Summary

## Data Availability

Data supporting the findings of this study was used under a license granted specifically for the current study and therefore is not publicly available according to the data protection regulations of Danish Data Protection Agency, Statistics Denmark and the Danish Health and Medicines Authority.

## Code availability

The statistical analyses were performed on the servers of Statistics Denmark and the coding is therefore not publicly available.

## References

[1] Hansen RP, Vedsted P, Sokolowski I, Sondergaard J, Olesen F (2011). Time intervals from first symptom to treatment of cancer: a cohort study of 2,212 newly diagnosed cancer patients. BMC Health Serv. Res..

[2] Shim J, Brindle L, Simon M, George S (2014). A systematic review of symptomatic diagnosis of lung cancer. Fam. Pract..

[3] Simon AE (2012). Knowledge of lung cancer symptoms and risk factors in the U.K.: development of a measure and results from a population-based survey. Thorax.

[4] Kennedy, M. P. T. et al. Lung cancer stage-shift following a symptom awareness campaign. *Thorax* **73**, 1128–1136 (2018). 10.1136/thoraxjnl-2018-211842 29950525

[5] Ironmonger L (2015). An evaluation of the impact of large-scale interventions to raise public awareness of a lung cancer symptom. Br. J. Cancer.

[6] Sele LM, Elnegaard S, Balasubramaniam K, Sondergaard J, Jarbol DE (2016). Lifestyle factors and contact to general practice with respiratory alarm symptoms-a population-based study. BMC Fam. Pract..

[7] Hamilton W, Peters TJ, Round A, Sharp D (2005). What are the clinical features of lung cancer before the diagnosis is made? A population based case-control study. Thorax.

[8] Lovgren M, Levealahti H, Tishelman C, Runesdotter S, Hamberg K (2008). Time spans from first symptom to treatment in patients with lung cancer–the influence of symptoms and demographic characteristics. Acta oncologica.

[9] Spiro, S. G., Gould, M. K. & Colice, G. L. Initial evaluation of the patient with lung cancer: symptoms, signs, laboratory tests, and paraneoplastic syndromes: ACCP evidenced-based clinical practice guidelines. *Chest* **132**, 149s–160s (2007). 10.1378/chest.07-1358 17873166

[10] Sele LM, Balasubramaniam K, Elnegaard S, Sondergaard J, Jarbol DE (2015). Lifestyle factors and experience of respiratory alarm symptoms in the general population. BMJ Open Respiratory Res..

[11] Balasubramaniam K, Ravn P, Larsen PV, Sondergaard J, Jarbol DE (2015). Specific and unspecific gynecological alarm symptoms–prevalence estimates in different age groups: a population-based study. Acta Obstetricia et. Gynecologica Scandinavica.

[12] Rasmussen S (2015). Overlap of symptoms of gastroesophageal reflux disease, dyspepsia and irritable bowel syndrome in the general population. Scand. J. Gastroenterol..

[13] Rasmussen S (2015). Specific and non-specific symptoms of colorectal cancer and contact to general practice. Fam. Pract..

[14] Rasmussen S (2018). Predictive values of upper gastrointestinal cancer alarm symptoms in the general population: a nationwide cohort study. BMC Cancer.

[15] Rasmussen, S. et al. Predictive value of colorectal cancer alarm symptoms in the general population. A nationwide cohort study. *Br. J. Cancer* **120**, 595–600 (2019). 10.1038/s41416-019-0385-x PMC6461905 30792531

[16] Cronin, K. A. et al. Annual report to the nation on the status of cancer, part I: National Cancer Statistics. *Cancer* **13**, 2785–2800 (2018). 10.1002/cncr.31551 PMC6033186 29786848

[17] Birring SS, Peake MD (2005). Symptoms and the early diagnosis of lung cancer. Thorax.

[18] Jensen HAR, Ekholm O, Davidsen M, Christensen AI (2019). The Danish health and morbidity surveys: study design and participant characteristics. BMC Med. Res. Methodol..

[19] Gjerstorff ML (2011). The Danish cancer registry. Scand. J. Public Health.

[20] Hovanec J (2018). Lung cancer and socioeconomic status in a pooled analysis of case-control studies. PLoS ONE.

[21] Sidorchuk A (2009). Socioeconomic differences in lung cancer incidence: a systematic review and meta-analysis. Cancer Causes Control.

[22] Rasmussen S (2014). The Danish Symptom Cohort: questionnaire and feasibility in the nationwide study on symptom experience and healthcare-seeking among 100 000 individuals. Int. J. Fam. Med..

[23] Whitaker KL, Scott SE, Winstanley K, Macleod U, Wardle J (2014). Attributions of cancer ‘alarm’ symptoms in a community sample. PLoS ONE.

[24] Elliott AM, McAteer A, Hannaford PC (2011). Revisiting the symptom iceberg in today’s primary care: results from a UK population survey. BMC Fam. Pract..

[25] Walabyeki J (2017). Experience of, awareness of and help-seeking for potential cancer symptoms in smokers and non-smokers: a cross-sectional study. PLoS ONE.

[26] Beckles MA, Spiro SG, Colice GL, Rudd RM (2003). Initial evaluation of the patient with lung cancer: symptoms, signs, laboratory tests, and paraneoplastic syndromes. Chest.

[27] Huggenberger, I. K. & Andersen, J. S. Predictive value of the official cancer alarm symptoms in general practice–a systematic review. *Danish Med. J.* **62**, (2015). 26050833

[28] Levitsky A (2019). Early symptoms and sensations as predictors of lung cancer: a machine learning multivariate model. Sci. Rep..

[29] Walter FM (2015). Symptoms and other factors associated with time to diagnosis and stage of lung cancer: a prospective cohort study. Br. J. Cancer.

[30] Shapley M, Mansell G, Jordan JL, Jordan KP (2010). Positive predictive values of >/=5% in primary care for cancer: systematic review. Br. J. Gen. Pract.: J. R. Coll. Gen. Practitioners.

[31] Jones R, Latinovic R, Charlton J, Gulliford MC (2007). Alarm symptoms in early diagnosis of cancer in primary care: cohort study using General Practice Research Database. BMJ.

[32] Ingeman ML, Christensen MB, Bro F, Knudsen ST, Vedsted P (2015). The Danish cancer pathway for patients with serious non-specific symptoms and signs of cancer-a cross-sectional study of patient characteristics and cancer probability. BMC Cancer.

[33] Baldwin DR, White B, Schmidt-Hansen M, Champion AR, Melder AM (2011). Diagnosis and treatment of lung cancer: summary of updated NICE guidance. BMJ.

[34] Pedersen CB (2011). The Danish civil registration system. Scand. J. Public Health.

[35] Torre LA, Siegel RL, Jemal A (2016). Lung cancer statistics. Adv. Exp. Med. Biol..

[36] Jensen VM, Rasmussen AW (2011). Danish education registers. Scand. J. Public Health.

[37] Baadsgaard M, Quitzau J (2011). Danish registers on personal income and transfer payments. Scand. J. Public Health.

[38] Petersson F, Baadsgaard M, Thygesen LC (2011). Danish registers on personal labour market affiliation. Scand. J. Public Health.

